# Imaging characteristics of hip joint microinstability: a case–control study of hip arthroscopy patients

**DOI:** 10.1007/s00256-024-04802-4

**Published:** 2024-10-05

**Authors:** Renuka M. Vesey, Andrew A. MacDonald, Matthew J. Brick, Catherine J. Bacon, Gen Lin Foo, Man Lu, Nicholas Lightfoot, Donna G. Blankenbaker, Rebecca M. Woodward

**Affiliations:** 1 Faculty of Medicine and Health Sciences, University of Auckland, Auckland, New Zealand; 2 Department of Radiology, North Shore Hospital, Auckland, New Zealand; 3 Orthosports North Harbour, Millenium Centre, Auckland, New Zealand; 4 Department of Anaesthesia and Pain Medicine, Middlemore Hospital, Auckland, New Zealand; 5 School of Medicine and Public Health, University of Wisconsin, Madison, WI USA; 6 Auckland Radiology Group (ARG), Auckland, New Zealand

**Keywords:** Hip microinstability, Hip instability, Hip laxity, Hip dysplasia

## Abstract

**Objectives:**

Hip microinstability is a clinical entity increasingly recognized and treated but challenging to diagnose with a lack of objective criteria. This study assessed the prevalence and diagnostic accuracy of different imaging findings for hip microinstability on radiograph and MR.

**Methods:**

A retrospective case–control study of 224 hips treated with arthroscopic surgery by a single orthopedic surgeon, 112 hips with clinical microinstability and 112 controls without. Pre-operative radiograph and MRI/MRA imaging were evaluated by two musculoskeletal radiologists to assess morphological parameters and imaging signs reportedly associated with hip microinstability.

**Results:**

Four imaging features reached significance as predictors of microinstability via three-step logistic regression: labral hyperplasia and decreased lateral center edge angle on MR (OR 2.45 and 0.93, respectively) and the absence of positive ischial spine sign and absence of osteophytes on radiographs (OR 0.47 and 0.28, respectively). Increased acetabular anteversion and absence of cam lesions were more likely in the microinstability group (*p* = 0.02 and 0.04, respectively), but not independent predictors. Labral tears, chondral loss, abnormal ligamentum teres, anterior capsule thinning, iliocapsularis to rectus femoris ratio, posterior crescent sign, cliff sign, and femoro-epiphyseal acetabular roof (FEAR) index were not associated with microinstabillity.

**Conclusion:**

Imaging features may be predictive of hip microinstability in some cases. Decreased LCEA, increased acetabular anteversion, and labral hyperplasia were associated with microinstability in this study, while many other published imaging findings were not. Imaging remains complementary, but not definitive, in the diagnosis of hip microinstability.

## Introduction

In recent years, new perspectives on hip pathology assessment and treatment have emerged, largely driven by rapid growth in the field of hip arthroscopy. Although historically considered a highly constrained and inherently stable joint, the hip is more biomechanically complex and dynamic than previously assumed [1–4]. There is increasing recognition that minute instability of the hip is detectable and clinically significant. Hip microinstability, a clinical entity defined as extraphysiologic hip joint motion that is symptomatic [5], is considered a cause of pain and dysfunction especially in the young athletic population and in females and is postulated to lead to premature osteoarthritis [1, 6, 7]. It is also identified as a substantial cause of poor outcomes following hip arthroscopic surgery or as an iatrogenic complication, potentially requiring revision surgery or conversion to hip replacement [8].

Accurate detection of hip microinstability pre-operatively is important, as the management and surgical technique involved in unstable hips differ considerably. This distinction is especially important in symptomatic hips with borderline hip dysplasia and possible femoroacetabular impingement (FAI), as an unstable hip could be managed with periacetabular osteotomy while a “stable” hip may be treated arthroscopically with correction of the impinging morphology [9]. Accurate diagnosis of hip microinstability allows for this to be optimally managed, including with hip preservation surgery where appropriate.

Currently, there are no objective diagnostic criteria for hip microinstability, with it being a composite diagnosis of clinical, imaging, and intraoperative findings [5, 10, 11]. A diagnostic tool by Khanduja et al. [5] was recently published; however, it is complex and reliant on expert clinical judgment. Multiple imaging features have been proposed as suggestive of hip microinstability, mostly indirect signs such as labral hyperplasia, iliocapsularis muscle hypertrophy, or anterior joint capsule thinning [12]. Many morphological and soft tissue factors thought to be relevant to or even causative of microinstability, such as acetabular dysplasia and labral tears, are also assessed in imaging studies [12].

Few primary studies have defined or evaluated the imaging characteristics of hip microinstability, especially in patients with clinically diagnosed microinstability. The purpose of this study is to determine the prevalence and diagnostic accuracy of a range of imaging findings, on radiograph and MR, that are predictive of hip microinstability to facilitate pre-operative management, including non-surgical therapies.

## Materials and methods

This study is a retrospective matched-pair case-control analysis evaluating pre-operative imaging findings of patients who underwent arthroscopic hip surgery, with and without a clinical diagnosis of hip microinstability. Informed consent was obtained from all participants. Patients provided written informed consent for their data to be collected for the purpose of ongoing research in the form of the Orthosports Prospective Surgical Outcomes Study. Ethics approval for this study was granted by the national ethics committee (New Zealand Health and Disability Ethics Committee) under Ethics Ref 17/NTA/269.

### Patient cohort

The database of surgical patients of one of the authors (MJB, orthopedic surgeon with a subspecialty interest in the hip) from his private practice in Auckland New Zealand was searched between August 2009 and May 2020. The identified patients had undergone primary arthroscopic hip surgery, were over 16 years old, and had symptoms refractory to non-operative management. Exclusion criteria included skeletally immature patients, those with lateral center edge angle (LCEA) < 20°, osteoarthritis on a radiograph (Tönnis grade > 1), history of previous hip surgery, or a history of prior diagnosed hip conditions (Perthes disease, slipped capital femoral epiphysis, avascular necrosis, acetabular fracture, femoral fracture, inflammatory arthritis). Participants with at least two of the following criteria formed the “microinstability group”:

1. Beighton score of 4 or more
2. Combined internal plus external hip rotation of 100 degrees or more on ipsilateral or contralateral hip
3. Positive hyperextension-external rotation hip test
4. Positive hip “dial” test
5. Intra-operative ease of hip joint distraction with standardized leg traction device

These were matched with cases without a diagnosis of microinstability (“control group”), in a 1:1 ratio, randomly selected with priority by sex, 5-year age bracket, and time of surgery (nearest quarter-year).

### Imaging

The demographic and clinical characteristics of the selected patients and the surgical procedures undertaken were retrieved from the surgeon’s prospective database. For all selected hips, an electronic search of regional PACS systems for pre-operative imaging was performed. Relevant pre-operative imaging, in the form of radiographs and MR imaging of the pelvis/hip, was retrieved for image analysis. MR examinations, including both conventional (non-arthrogram) MRI and MR arthrogram (MRA), with MR acquired on both 1.5 T and 3.0 T systems, and included examinations with both large field-of-view and small field-of-view (FOV < 18 cm) imaging of the symptomatic hip. Some cases included additional imaging through the femoral condyles on MR. Imaging studies were obtained from a range of providers and imaging systems in New Zealand.

### Image analysis

All MR examinations were independently assessed by two experienced musculoskeletal radiologists (RW and DB), both with over 20 years of experience in musculoskeletal radiology, and imaging findings averaged. All radiographs were assessed by a single observer (RW). Imaging was reviewed in the chronological order that the MR studies were performed, with both readers blinded to the clinical diagnosis. For assessment of intraobserver reliability, approximately one-third of MR and radiograph examinations were re-measured 2 weeks later by a single observer (RW). Imaging parameters were assessed according to techniques described in Tables 1 and 2.

**Table 1:** Methods for radiographs

Feature	Description	Reference
Lateral center edge angle	Vertical line through the FHC perpendicular to inter tear drop line and a line drawn from FHC to the lateral edge of the weight bearing surface (sourcil)	Tibor 2013 [13]
Acetabular index	Angle formed by a transverse axis of pelvis and a line through the medial and lateral edge of the acetabular roof	Mascarenhas 2019 [14]
Femoral neck shaft angle	Angle formed by a line extending through FHC along axis of femoral neck with an intersecting line drawn along axis of femoral shaft	Beltran 2013 [15]
Shenton’s line	Interrupted if the inferior femoral head neck contour and superior border of the obturator foramen do not form a smooth arc	Mascarenhas 2019 [14]
Cross over sign	Cranial prominence of the anterior acetabular wall with a “figure of 8” configuration	Tannast 2007 [16]
Posterior wall sign	On best fit circle FHC lies lateral to the posterior wall	Reynolds 1999 [17]
Ischial spine sign	Ischial spines project medial to pelvic rim	Tannast 2007 [16]
Ischial spine	Ischial spine sign $\ge$ 5 mm	
Cliff sign	Positive if steep drop off at femoral head neck junction so a circle over the femoral head is incompletely filled in this region	Packer 2018 [18]
The femoro-epiphyseal acetabular roof (FEAR) index	Angle between line from the most lateral to the most medial point of the straight central third of the femoral head physeal scar and a line from the most medial and lateral points of the sourcil	Wyatt 2017 [19]
Cam lesion	Anterosuperior osseous convexity at the femoral head neck junction	Mascarenhas 2019 [14]
Joint space narrowing	Superior narrowing < 2 mm	
Osteophytes	Presence	
Pelvic tilt	Distance between sacrococcygeal joint < 3 cm or > 5 cm	Tannast 2007 [16]
Rotation	> 1 cm malalignment of the middle of the sacrococcygeal joint and pubic symphysis	Tannast 2007 [16]

*FHC*, femoral head center

**Table 2:** Methods for magnetic resonance imaging

Feature	Description	Reference
Lateral center edge angle	On mid coronal slice, vertical line through the FHC perpendicular to inter tear drop line and a line drawn from FHC to the lateral edge of the weight bearing surface (sourcil)	Beltran 2013 [15]
Horizontal acetabular sector angle	On axial image at level of FHC, angle between lines from anterior lip of acetabulum to FHC and posterior lip of acetabulum	Beltran 2013 [15]
Anterior acetabular sector angle	On axial image at level of FHC, angle between lines from anterior lip of acetabulum to FHC and a horizontal line	Beltran 2013 [15]
Posterior acetabular sector angle	On axial image at level of FHC, angle between lines from posterior lip of acetabulum to FHC and a horizontal line	Beltran 2013 [15]
Acetabular anteversion	On axial image at level of FHC, angle between the lines connecting the anterior to posterior acetabular rim and the sagittal plane	Suter 2015 [20]
Cranial acetabular retroversion	Presence of retroversion of acetabular margin just below superior joint line	Suter 2015 [20]
Femoral torsion	Angle between axis of femoral neck and the posterior margin of the femoral condyles	Reikeras 1983 [21]
Ratio iliocapsularis to rectus femoris width and thickness	On axial image at level of FHC, muscle thickness measured along radial line from FHC, width measured perpendicular to thickness	Haefali 2015 [22]
Capsule thickness — superior iliofemoral ligament	On mid coronal image, thickness of mid portion of superior iliofemoral ligament medial to zona orbicularis	Suter 2015 [20]
Capsule thickness — inferior iliofemoral ligament	Thickness of mid portion of inferior iliofemoral ligament on sagittal (with whole length of capsule visualized)	Suter 2015 [20]
Posterior crescent sign	Fluid separating cartilage surfaces around full circumference of posterior femoral head	MacDonald 2023 [23]
Abnormal ligamentum teres	Increased signal within ligament, thickened, attenuated or elongated ligamentum teres	Blankenbaker 2012 [24]
Labral tear and high grade chondral damage	Presence and quadrant	Fontana 2012 [25]
Labral hyperplasia	$\ge$ 10 mm	
High grade acetabular cartilage damage	Presence, quadrant, peripheral or central, size ≥ 10 mm	
Acetabular subchondral reactive change	Presence, peripheral or central, size ≥ 10 mm	
High grade femoral cartilage damage	Presence, weightbearing surface, size ≥ 10 mm	
Subchondral reactive change in femoral head	Presence, weightbearing surface, size ≥ 10 mm	
Osteophytes	Presence	

*FHC*, femoral head center

### Statistical analysis

Statistical analysis was performed using SPSS Version 27 (IBM Corporation, Armonk, NY, USA) and NCSS Version 21.0.3 (NCSS LLC, Kaysville, UT, USA). All statistical tests were two-tailed with the level of significance to reject the null hypothesis set at *p* ≤ 0.05. Statistical comparisons between groups were made with the Mann–Whitney *U*-test for continuous variables and the Fisher’s exact test or chi-square test with an appropriate continuity correction for binomial variables. Measured data are reported as “median (interquartile range)” for continuous parameters and “number (percent)” for discrete or binomial parameters.

Intraobserver and interobserver reliability of continuous parameters was assessed with intraclass correlation coefficient (ICC), one-way random effects model, and two-way random effects model using a consistency definition, respectively; Cohen’s weighted kappa (κ) measured discrete or binomial parameters. Reliability results were reported as an average (95% confidence interval). ICC were interpreted as follows: values < 0.5 were considered poor agreement, between 0.5 and 0.75 for moderate agreement, between 0.75 and 0.9 for good agreement, and values > 0.9 were considered excellent agreement. *κ* were interpreted as follows: values ≤ 0 indicate no agreement, 0.01–0.20 as none to slight agreement, 0.21–0.40 as fair agreement, 0.41–0.60 as moderate agreement, 0.61–0.80 as substantial agreement, and 0.81–1.00 as almost perfect agreement.

Multivariate regression models were generated using logistic regression backwards elimination, to calculate likelihood ratios for imaging features as predictors of hip microinstability. Imaging features included as candidate variables in the radiograph model were as follows: osteophytes, ischial spine $\ge$ 5 mm, cam lesion, LCEA, and acetabular index. Imaging features included as candidate variables in the MR model were as follows: LCEA, acetabular anteversion, labral hyperplasia, and the presence of anteroinferior labral tear. Model “goodness of fit” statistics with Nagelkerke R Square and Hosmer and Lemeshow test were calculated. Regression model results were reported as “Odds Ratio (OR); 95% confidence interval”.

## Results

This study included a total of 224 hips (112 hips in each group), derived from 211 patients (26 bilateral hips: 2 in the microinstability group and 24 in the control group). The total study population had a median age of 28.9 years, was 97% female, and had a median BMI of 24.2 kg/m^2^. There were no significant differences between groups in demographic characteristics; these and the main surgical procedures performed in each group are reported in Table 3.

**Table 3:** Demographic and surgical characteristics

Characteristic	Microinstability (*n* = 112)	Control (*n* = 112)	*p*-value
Female sex	109 (97)	109 (97)	1.00
Age (years)	27.3 (22.9–36.1)	27.5 (21.2–37.0)	0.94
Body mass index (kg/m^2^)	23.4 (21.6–26.7)	23.4 (21.8–26.0)	0.94
Capsular repair	2 (2)	85 (76)	** < 0.001**
Capsular plication	110 (98)	2 (2)	** < 0.001**
Acetabular microfracture	6 (5)	10 (9)	0.44
Labral repair	89 (80)	92 (82)	0.74
Femoral osteoplasty	78 (70)	94 (84)	0.02

Note values presented as number (percent) or median (interquartile range) as appropriate

Bolded *p*-values indicate statistical significance

Radiographs were available for 215 (96%) hips in total, 109 (97%) in the microinstability group, and 102 (91%) in the control group. Of these, 16 (7%) radiographs were rotated, and 126 (59%) demonstrated excessive pelvic tilt - 120 with increased pelvic tilt. Of the 211 (94%) MR hip studies available, 93 (44%) were conventional MRI (non-arthrogram) and 118 (56%) were MRA. MR imaging of the hip included 150 (71%) studies with small field-of-view (FOV < 18 cm) sequences, 198 (94%) with axial-oblique sequences, and 95 (45%) with radial sequences, while 48 (21%) included axial femoral condyle imaging at the knee. Imaging availability, quality, and type were not statistically significantly different between groups.

Labral tears were observed on MR in 197 (93%) of hips across both groups, with nearly identical rates between the microinstability and control groups (see Table 4), with 196 (99.5%) of these (across both groups) involving the anterosuperior labrum. Of those that had a labral tear, 40 (39%) of these in the microinstability group and 27 (28%) of these in the control group also involved the anteroinferior labrum (extending inferior to 3:00), with no significant difference between groups (*p* = 0.10). Labral tears involving the posterior labrum were uncommon, with tears in these locations in 9 (9%) of the microinstability group and 10 (10%) of the control group (*p* = 1.00).

**Table 4:** MRI/MRA imaging features

Imaging feature	Microinstability (*n* = 109)	Control (*n* = 102)	*p*-value
Lateral center edge angle (°)	28.5 (26.2–31.4)	30.3 (26.7–33.5)	**0.02**
Horizontal acetabular sector angle (°)	157.5 (151.5–164.7)	156.7 (150.6–164.5)	0.70
Anterior acetabular sector angle (°)	59.2 (55.5–64.0)	61.2 (57.2–64.4)	0.10
Posterior acetabular sector angle (°)	99.0 (92.7–103.1)	95.8 (90.1–100.9)	0.07
Acetabular anteversion (°)	20.2 (17.2–23.8)	18.8 (15.9–22.0)	**0.02**
Cranial acetabular retroversion	20 (19)	25 (25)	0.32
Femoral torsion (*n* = 48)	19.6 (12.1–27.9)	15.2 (11.5–21.7)	0.27
IC to RF width ratio	0.96 (0.83–1.18)	0.93 (0.79–1.14)	0.60
IC to RF thickness ratio	1.03 (0.88–1.20)	1.08 (0.88–1.22)	0.97
Anterior capsule thickness — superior (mm)	4.0 (3.0–4.0)	3.5 (3.0–4.5)	0.25
Anterior capsule thickness — inferior (mm)	3.5 (3.0–4.5)	3.5 (3.0–4.5)	0.80
Labral tear	101 (93)	96 (94)	0.79
Labral hyperplasia	22 (20)	9 (9)	**0.03**
High-grade chondral loss — acetabulum	24 (22)	24 (24)	0.87
High-grade chondral loss — femoral head	9 (8)	12 (12)	0.49
Chondral loss over central femoral head	6 (6)	8 (8)	0.59
Cam lesion	8 (7)	10 (10)	0.62
Posterior crescent sign	4 (4)	1 (1)	0.37
Abnormal ligamentum teres	28 (25)	28 (27)	0.88

*IC to RF*, iliocapsularis to rectus femoris

Note values presented as number (percent) or median (interquartile range) as appropriate

Bolded *p*-values indicate statistical significance

On average, the measured morphological parameters on both radiograph and MR (LCEA, HASA, AASA, PASA, acetabular anteversion, femoral torsion) were within normal limits across both the microinstability and control groups (see Tables 4 and 5), with the medians and interquartile ranges all above the commonly reported thresholds of abnormality (LCEA < 20°; HASA < 140°; AASA < 50°; PASA < 90°; acetabular version > 25° or < 10°, femoral torsion < 5° or > 25°) [19, 26].

**Table 5:** Radiographic imaging features

Imaging feature	Microinstability group (*n* = 111)	Control Group (*n* = 104)	*p*-value
Lateral center edge angle (°)	29.7 (25.6–33.2)	31.7 (27.4–36.3)	**0.004**
Acetabular index (°)	4.5 (1.4–8.1)	3.0 (1.0–6.2)	0.06
Femoral neck-shaft angle (°)	132.4 (129.7–137.6)	132.7 (129.4–135.3)	0.47
Shenton line disrupted	110 (100)	101 (98)	0.23
Crossover sign	41 (37)	40 (39)	0.89
Posterior wall sign	32 (29)	39 (38)	0.25
Ischial spine sign	54 (49)	54 (52)	0.68
Ischial spine > 5 mm	23 (21)	39 (38)	**0.01**
Cliff sign	46 (42)	35 (34)	0.21
FEAR index (°)	− 13.5 (− 20.5 to − 5.4)	− 15.0 (− 22.1 to − 6.3)	0.69
Cam lesion (present)	4 (4)	12 (12)	**0.04**
Joint space narrowing	3 (3)	2 (2)	1.00
Osteophytes (present)	4 (4)	14 (14)	**0.01**

*FEAR*, femoro-epiphyseal acetabular roof

Note values presented as number (percent) or median (interquartile range) as appropriate

Bolded *p*-values indicate statistical significance

Six imaging features showed a statistically significant association with the microinstability group in comparison to the control group, see Tables 4 and 5: lower LCEA on both radiograph (*p* = 0.004) and MR (*p* = 0.02); increased acetabular anteversion on MR (*p* = 0.02); higher rate of labral hyperplasia on MR (*p* = 0.03); lower rate of osteophytes on radiograph (*p* = 0.01); lower rate of cam lesion on radiograph (*p* = 0.04); and lower rate of positive ischial spine (≥ 5 mm) on radiograph (*p* = 0.01).

Intraobserver assessment showed almost perfect agreement for measurement of both LCEA and acetabular index/anteversion on both radiograph and MR (*κ* = 0.95–0.97 for all), see Table 6. Interobserver reliability for MR imaging findings was more variable, with a substantial agreement for measurement of LCEA (ICC = 0.81) and acetabular anteversion (ICC = 0.79), but a fair or moderate agreement for most other findings (*κ* = 0.26–0.54), except for the finding of abnormal ligamentum teres where there was no agreement (*κ* =  − 0.01).

**Table 6:** Reliability of imaging features

	Imaging feature	ICC or *κ* (95% CI)
**Intraobserver reliability**	Radiograph, lateral center edge angle	0.96 (0.94–0.98)
	Radiograph, acetabular index	0.96 (0.92–0.98)
	MR, lateral center edge angle	0.95 (0.93–0.97)
	MR, acetabular anteversion	0.97 (0.95–0.98)
**Interobserver reliability**	Lateral center edge angle	0.83 (0.78–0.87)
	Acetabular anteversion	0.79 (0.72–0.84)
	Labral hypertrophy	0.26 (0.08–0.44)
	Labral tear (any location)	0.26 (0.00–0.51)
	Chondral loss over central femoral head	0.34 (0.11–0.57)
	Cam lesion	0.54 (0.32–0.77)
	Abnormal ligamentum teres	− 0.01 (− 0.01–0.31)
	Osteophytes	0.47 (0.27–0.66)

Note interobserver reliability included magnetic resonance (MR) imaging only; intraclass correlation coefficient (ICC) was used for continuous parameters and Cohen’s weighted kappa (*κ*) for discrete or binomial parameters with 95% confidence intervals

The multivariate regression models yielded four imaging features which reached significance as predictors of hip microinstability (see Table 7). Labral hyperplasia on MR was positively associated with hip microinstability (OR = 2.45; *p* = 0.04). Three imaging features were negatively associated with hip microinstability: LCEA on MR (OR = 0.93 per degree; *p* = 0.03), the presence of osteophytes on a radiograph (OR = 0.28; *p* = 0.03), and ischial spine sign (≥ 5 mm) on a radiograph (OR = 0.47; *p* = 0.02).

**Table 7:** Logistic regression model of predictors of microinstability

Imaging feature	Odds ratio (95% CI)	*p*-value
Radiograph, osteophytes	0.28 (0.09–0.91)	**0.03**
Radiograph, cam lesion	0.32 (0.10–1.06)	0.06
Radiograph, ischial spine > 5 mm	0.47 (0.25–0.88)	**0.02**
MR, lateral center edge angle (per °)	0.93 (0.88–0.99)	**0.03**
MR, labral hyperplasia	2.45 (1.05–5.72)	**0.04**

*CI*, confidence interval; *MR*, magnetic resonance

MR: Nagelkerke R Square = 0.069; Hosmer and Lemeshow test *p* = 0.14

Radiograph: Nagelkerke R Square = 0.102; Hosmer and Lemeshow test *p* = 0.90

Bolded *p*-values indicate statistical significance

## Discussion

Comprehensive assessment of radiographic and MR imaging of the hip in this study found only six imaging features associated with a diagnosis of hip microinstability: decreased LCEA, increased acetabular anteversion, and the presence of labral hyperplasia on MR; and the absence of osteophytes, absence of cam lesion, and absence of ischial spine ≥ 5 mm on radiographs. Four of these were independent predictors of microinstability. Many other imaging features reportedly associated with hip microinstability were not associated in this cohort, including the location of labral tears, cartilage loss, abnormal ligamentum teres, anterior capsule thinning, iliocapsularis to rectus femoris (IC to RF) ratio, posterior crescent sign, cliff sign, and femoro-epiphyseal acetabular roof (FEAR) index.

This study is one of few published studies comprehensively examining both radiographs and MR in patients with a clinically established diagnosis of hip microinstability. It is also thought to be the largest, with a cohort of 109 positive hips with complete imaging available. The existing literature includes case reports, small single-group descriptive studies, and small cohort studies of patients with hip microinstability, as well as research on hip arthroscopy patients or other joint pathology (without distinguishing a hip microinstability cohort) [12]. Six published studies of a substantial study population (*n* = 21–50) have specifically evaluated a cohort with hip microinstability [9, 18–20, 27, 28], with the latter half evaluating MR imaging — the remainder evaluated radiographs only.

Decreased LCEA in association with hip microinstability is an important finding of this study, as LCEA is one of the few parameters routinely assessed on imaging of the hip in clinical practice, and it has a plausible etiological connection with hip microinstability. A decreased LCEA corresponds to comparatively less acetabular coverage, which is the key structural element in hip dysplasia; biomechanical and clinical studies have demonstrated the degree of instability in dysplastic hips is inversely proportional to LCEA [20, 29–32]. It follows that microinstability could be a feature in cases with mild acetabular under-coverage. This result concurs with other research that has reported an association between microinstability and hip dysplasia [12] and supports the inclusion of dysplasia as a major factor in the recently published diagnostic tool by Khanduja et al. [5].

In this study, although cases of hip microinstability had a smaller LCEA on average than those without, the LCEA was in most cases within the normal range of > 25°. This in part reflects the exclusion of those with hip dysplasia from this study population undergoing hip arthroscopy. Nevertheless, this is concordant with other published studies which have found that most patients diagnosed with hip microinstability have an LCEA in the normal range [12]. Some cases of hip microinstability with a normal LCEA may also reflect reduced anterior or posterior coverage, as acetabular undercoverage is a complex three-dimensional entity not always completely represented by a single measure of lateral coverage. Although we found increased acetabular anteversion in the microinstability group, there was no difference in the anterior and posterior sector angles (AASA and PASA) between groups. This differs from Hatem et al. [27] who found AASA to be smaller in hip microinstability compared to controls.

Abnormal acetabular version and femoral torsion are reported to be associated with hip microinstability [4]. It is suggested that increased acetabular anteversion (version > 25°) and/or increased femoral torsion (antetorsion) predisposes to “anterior instability” by reducing the relative degree of functional anterior acetabular coverage and potentially inducing FAI via bone contact with the prominent posterior rim [4]. In contrast, acetabular retroversion (version < 10°) or decreased femoral torsion/version (retrotorsion) is said to induce “posterior instability” as a consequence of FAI at the prominent anterior rim [4]. The concurrence of FAI and joint instability has been increasingly recognized in recent years [33]. In line with emerging literature, our study found a small but statistically significant increased acetabular anteversion in microinstability compared to controls, although this was not predictive in the logistic regression model. Acetabular retroversion was not found to be associated with microinstability in this study by direct measurement on MR or indirectly on radiograph via signs of acetabular retroversion (crossover sign, posterior wall sign, ischial spine sign). The assessment of femoral torsion on MR of the hip requires additional axial femoral condyle imaging at the knee and is relatively recent in its introduction into clinical practice. In our study, only a small subset of patients imaged in later years included this, and there was no significant difference between groups.

**Fig. 1:**
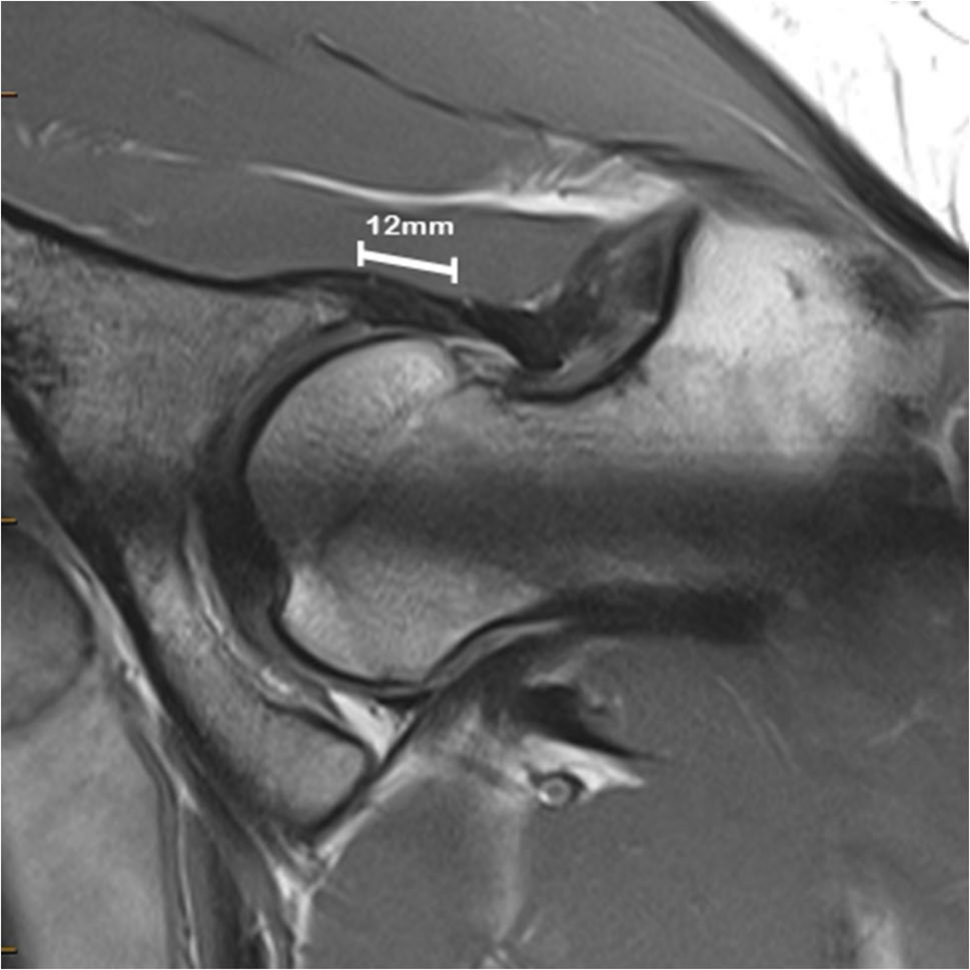
Radial PD MR image in a 21-year-old female with clinical microinstability, demonstrating elongated labrum superior labrum

Labral hyperplasia, see Fig. 1, in association with hip microinstability is one of the most important positive findings of this study because it has been proposed by some authors as a specific marker of hip microinstability. This is the third imaging study to evaluate labral hyperplasia in microinstability, with Suter et al. [20] finding no association with labral size but more recently Andronic et al. [28] reported a positive association (using a labral height-to-length ratio of ≤ 0.8). The evidence for this reported association is otherwise derived from surgical studies of arthroscopy findings in patients mostly with dysplasia or FAI (not specifically hip microinstability) [34–36]. This study finds labral hyperplasia (of > 10 mm) to be significantly more common in, although not exclusive to, cases of hip microinstability with a relatively high odds ratio and fair interobserver reliability. Labral hyperplasia has been postulated to be a reactive or adaptive response to increased rim stresses in the setting of hip instability [37].

**Fig. 2:**
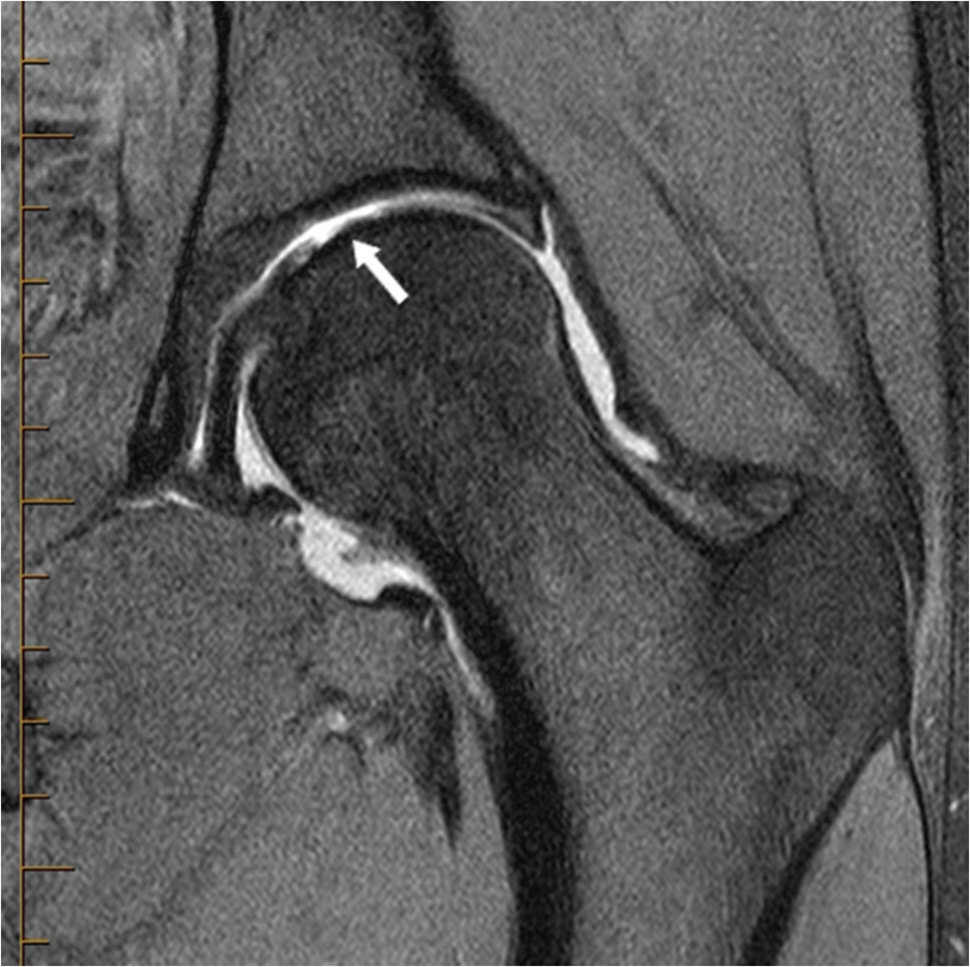
Coronal fat saturated PD MRA image in a 27-year-old female with clinical microinstability, demonstrating central femoral head cartilage damage

The location of labral tears and/or cartilage loss was not associated with hip microinstability in this study. Most labral tears were located anterosuperior with variable extension anteroinferiorly below 3 o’clock. The localization of labral tears on MRI may not be directly comparable with that used at arthroscopy [38]. It is noted that slight differences in adjustments for pelvic tilt can change the quadrant radiologists assign tears to. Chondrolabral injuries are reported to be common in hip microinstability, with certain locations or morphological configurations said to be characteristic, including labral tears in the direct anterior location (3–4 o’clock), injuries at the chondrolabral junction especially with an “inside out” morphology, and chondral loss of the central femoral head (around the fovea capitis), Safran [10], see Fig. 2. However, these findings are mostly from studies of intraoperative surgical findings; the few imaging studies of these report conflicting results [12], and none was included in the recently published diagnostic tool by Khanduja et al. [5]. It is possible that the spatial resolution of MR, even on modern 3 T MRI or MRA, is not sufficient for the detection of these subtle lesions, in comparison to the high level of visual and tactile detail offered by intraoperative assessment.

With regard to labral tear prevalence, tears were extremely common across both groups in this study (93%). Cross-sectional studies of labral tears on MR have reported highly variable prevalence rates, but most have reported labral tears to be present in a majority of asymptomatic people, one systematic review reported this to be 68% [39]. This calls into question the relevance and specificity of labral tears as an isolated imaging finding in general.

The function and significance of the ligamentum teres are debated; however, it is increasingly recognized as a secondary stabilizer of the hip joint, and biomechanical and surgical studies have suggested ligamentum teres injuries are associated with joint instability [40, 41]. While complete tears of the ligamentum teres are relatively rare, with the prevalence of up to 1.5% reported at arthroscopy [42], partial tears and other abnormalities such as hyperplasia are commonly reported at arthroscopy. Abnormal ligamentum teres on MR was not associated with hip microinstability in this study.

Thinning of the anterior joint capsule is another imaging finding not associated with hip microinstability in this study but is commonly reported as a diagnostic finding in the literature [12]. A thin anterior capsule (< 3 mm) on MR was included as a “minor factor” in the recently published diagnostic tool by Khanduja et al. [5]. Several small studies have reported an association between a thin anterior capsule and decreased LCEA/hip dysplasia, generalised laxity/joint hypermobility, or a diagnosis of hip microinstability [43–45]. However, the largest study to assess this by Suter et al. [20] with 50 positive hips on traction MRA is concordant with this study, finding no association. It is noted that there is a high degree of variability in the technique and anatomic location for assessing capsule thickness on MR [12]. In this study, joint capsule thickness was measured at both a superior site and inferior site corresponding to the superior and inferior bands of the iliofemoral ligament as described by Suter et al. [20], but in both cases, there was no significant difference between groups.

**Fig. 3:**
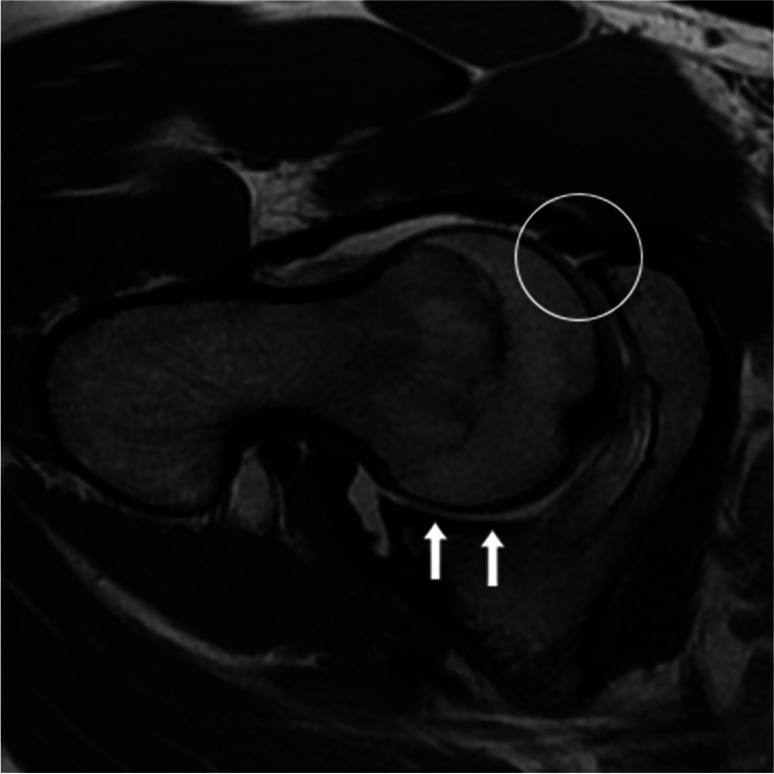
Oblique axial PD MRA image in a 27-year-old female with clinical microinstability, demonstrating a posterior crescent

**Fig. 4:**
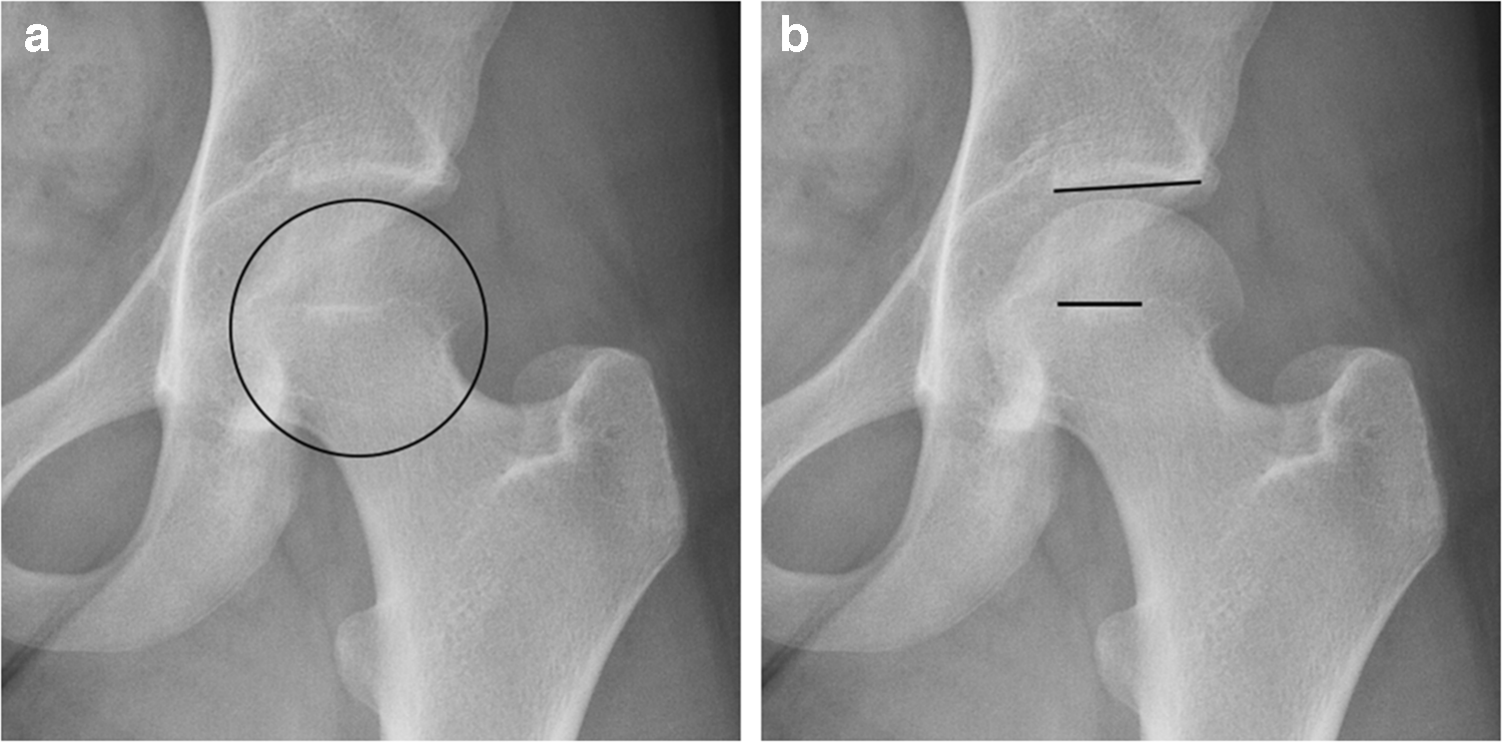
Radiograph in a 17-year-old female with clinical microinstability demonstrating a positive cliff sign (**A**) and a FEAR index of +5 degrees (**B**)

Four other novel imaging signs reported in the literature to be predictive and relatively specific for hip microinstability were not associated in this study: IC to RF ratio, posterior crescent sign, cliff sign, and FEAR index. An increased IC to RF ratio denotes relative hyperplasia of the iliocapsularis muscle, a periarticular muscle with capsular attachment to the anteromedial hip joint capsule which is thought to be a dynamic stabilizer in unstable hips [46]. One study reported an increased IC to RF ratio to be associated with hip dysplasia [22]; however, it was not associated with hip microinstability in a recent imaging study by Andronic et al. [28]. The posterior crescent sign, as demonstrated in Fig. 3, represents a crescenteric accumulation of fluid or intra-articular contrast in the postero-inferior joint space of the hip on MRI or MRA [23] and is said to be diagnostic of hip microinstability [47]; however, few studies have formally investigated its diagnostic accuracy [23, 48, 49]. The cliff sign and increased FEAR index (> 5°), as shown in Fig. 4, are both imaging findings on radiograph reported to be predictive of hip microinstability and were included in the recently published diagnostic tool by Khanduja et al. [5], with an increased FEAR index (> 5°) considered a “major factor.”

In this study, imaging findings related to both degenerative joint disease and FAI were mixed. Both osteophytes and a cam lesion on radiographs were (separately) significantly more common in the control group than those with hip microinstability; in the logistic regression model, osteophytes were negatively associated with hip microinstability, while cam lesions did not reach statistical significance. However, on MR neither of these features, nor the presence of high-grade chondral loss, were significantly different between groups. The clinical significance of these findings is therefore unclear. The relationship between a cam lesion and hip microinstability is complex, as FAI has been increasingly recognized as a cause of a subset of microinstability, as described above, and the presence of a cam lesion is listed as a “minor factor” in the diagnostic tool by Khanduja et al. [5].

It is important to consider the mixed reliability of these imaging findings when assessed by radiologists. The measurement of LCEA and acetabular anteversion showed almost perfect intraobserver agreement on both radiograph and MR (ICC 0.95–0.97) and substantial interobserver agreement on MR (ICC 0.83 and 0.79 respectively), confirming that these important morphological measurements are reproducible and likely to be reliable when assessed by musculoskeletal radiologists. However, the other imaging findings on MR showed only fair or moderate agreement, except for abnormal ligamentum teres which showed no agreement, when assessed by experienced musculoskeletal radiologists. This is consistent with the published literature that has documented that many of these findings can be challenging to accurately detect on MR with limited or even poor reliability; diagnosis of ligamentum teres tear, especially chronic and partial tears, may be challenging on MR with poor sensitivity and specificity [24, 50, 51].

A limitation of this study is that the classification of cases into the microinstability cohort relied on the recorded clinical diagnosis by the single treating surgeon, with no diagnostic gold standard in existence. The diagnosis of hip microinstability is notoriously challenging and subjective, and there remains no single objective diagnostic marker. Diagnostic criteria and thresholds have also changed over the course of the inclusion period of this study as knowledge of this condition has evolved. The international expert consensus paper outlining the diagnosis of hip microinstability by Khanduja et al. [5] includes a range of major and minor criteria with no definitive diagnostic threshold, and it was published after patient participation in this study. In any case, this study is reflective of current clinical practice. It is also important to note that this study excluded patients with dysplasia (LCEA < 20°) by virtue of these cases not routinely being treated by hip arthroscopy in this surgeon’s usual practice.

In conclusion, in the appropriate clinical context, imaging may be predictive of hip microinstability. Decreased LCEA, increased acetabular anteversion, and labral hyperplasia were positively associated with microinstability in this study, while many other published imaging findings were not. Acknowledging the observer variation and lack of threshold values, imaging remains complementary but not definitive to the diagnosis of hip microinstability.

## Data Availability

Data are available upon reasonable request.
